# *Pseudomonas aeruginosa*-driven airway dysbiosis and machine learning prediction of acute exacerbations in non-cystic fibrosis bronchiectasis: a microbial-inflammatory signature approach

**DOI:** 10.1186/s12890-025-03892-7

**Published:** 2025-09-01

**Authors:** Wen-Wen Wang, Yu-Han Wang, Jian Xu, Yuan-Lin Song, Jin-Fu Xu

**Affiliations:** 1 Department of Respiratory and Critical Care Medicine, Zhongshan Hospital, Fudan University, Shanghai, China; 2 Department of Respiratory and Critical Care Medicine, Shanghai Pulmonary Hospital, Tongji University, Shanghai, China; 3 Shanghai Institute of Infectious Disease and Biosecurity, Fudan University, Shanghai, China; 4 Department of Respiratory and Critical Care Medicine, Huadong Hospital, Fudan University, Shanghai, China

**Keywords:** Bronchiectasis, Lung microbiome, Pseudomonas aeruginosa, Machine learning, XGBoost, SHAP analysis

## Abstract

**Background:**

While *Pseudomonas aeruginosa* (PA) colonization is linked to poor outcomes in bronchiectasis, emerging evidence suggests that microbial community collapse—marked by diversity loss and depletion of commensal taxa—may better reflect disease progression than pathogen load alone. This study investigates whether airway microbiota dysbiosis driven by PA colonization induces ecological fragility and evaluates the predictive utility of integrating microbial diversity indices with systemic inflammation markers to forecast 1-year acute exacerbation risk using interpretable machine learning.

**Methods:**

Bronchoalveolar lavage fluid (BALF) samples from 23 patients (8 PA-colonized, 15 non-colonized) underwent 16 S rRNA gene sequencing. Microbial diversity and taxonomic composition were analyzed. An eXtreme Gradient Boosting (XGBoost) model with SHapley Additive exPlanations (SHAP) analysis was constructed to assess exacerbation risk, focusing on microbial and inflammatory markers.

**Results:**

PA-colonized patients (P1) exhibited significantly worse clinical severity than non-colonized patients (P2), with higher Bronchiectasis Severity Index scores (8.38 vs. 4.33, *P* < 0.01), poorer quality-of-life (SGRQ: 35.75 vs. 22.79; CAT: 24.00 vs. 16.26, *P* < 0.01), and elevated dyspnea (mMRC: 1.62 vs. 0.95, *P* < 0.05). P1 also had more acute exacerbations annually (retrospective: 3.00 vs. 1.20; prospective: 3.75 vs. 0.80, *P* < 0.05–0.001). Notably, P1 exhibited significantly reduced alpha diversity compared to P2 (Shannon index: 1.96 vs. 3.47; Simpson index: 0.46 vs. 0.77, *P* < 0.05). Weighted UniFrac PCoA revealed distinct clustering between groups (R²=0.162, *P* < 0.05). The XGBoost model, integrating microbial taxa relative abundances, alpha diversity indices, and inflammatory markers demonstrated robust performance in predicting 1-year acute exacerbation risk (AUC = 0.85). SHAP analysis identified the microbial diversity, rather than *Pseudomona* abundance was the most influential predictor of exacerbation risk.

**Conclusions:**

PA colonization disrupts airway microbial diversity and outcompetes commensal species in bronchiectasis, yet our XGBoost model reveals that ecological resilience—not pathogen load—best predicts exacerbation risk when integrated with inflammatory markers. This paradigm shift from pathogen-centric to ecosystem-driven risk assessment provides an actionable framework for personalized management and antibiotic stewardship in chronic airway diseases.

**Supplementary Information:**

The online version contains supplementary material available at 10.1186/s12890-025-03892-7.

## Background

Bronchiectasis, a chronic respiratory disease characterized by irreversible bronchial dilation and recurrent infections [1], arises from dynamic interplay between host immunity, environmental triggers, and airway microbiota perturbations [2]. Although *Pseudomonas aeruginosa* (PA) colonization is a well-established prognostic marker—associated with frequent exacerbations and accelerated lung function decline [3] —the mechanistic links between PA-driven microbial dysbiosis and clinical deterioration remain elusive. Emerging evidence posits that PA’s virulence extends beyond direct tissue damage to include destabilization of the airway microbial ecosystem through competitive exclusion of commensal taxa and suppression of community diversity [4], However, conventional severity assessments like the Bronchiectasis Severity Index (BSI) focus on clinical parameters and pathogen load but neglect microbiota-derived ecological resilience metrics [5–7], impeding the development of targeted therapies and precision risk stratification.

Recent studies highlight the role of PA-dominated microbiomes in driving systemic inflammation, and reduced microbiota diversity [8, 9]. Integrative analyses of microbiota and inflammatory markers have identified distinct endotypes, with neutrophilic inflammation profiles aligning with PA predominance and heightened exacerbation risk [10], yet few studies integrate these insights with inflammatory and clinical data to refine prognostic frameworks. Machine learning offers a promising solution by capturing non-linear relationships and feature interactions in high-dimensional datasets [11], but its application in bronchiectasis remains underexplored.

Here, we hypothesize that PA colonization induces ecological fragility (reduced diversity) in the bronchoalveolar niche, and that integrating microbial resilience indices with systemic inflammation markers can outperform existing models in exacerbation risk prediction. To test this, we profile airway microbiota via 16 S rRNA gene sequencing in PA-colonized versus non-colonized patients and develop an interpretable eXtreme Gradient Boosting (XGBoost) machine learning framework—a method increasingly validated for complex biomedical data [12]—to identify predictive signatures of microbial-inflammatory synergy, independent of clinical severity scores.

## Methods

### Participant enrollment and study design

**Fig. 1:**
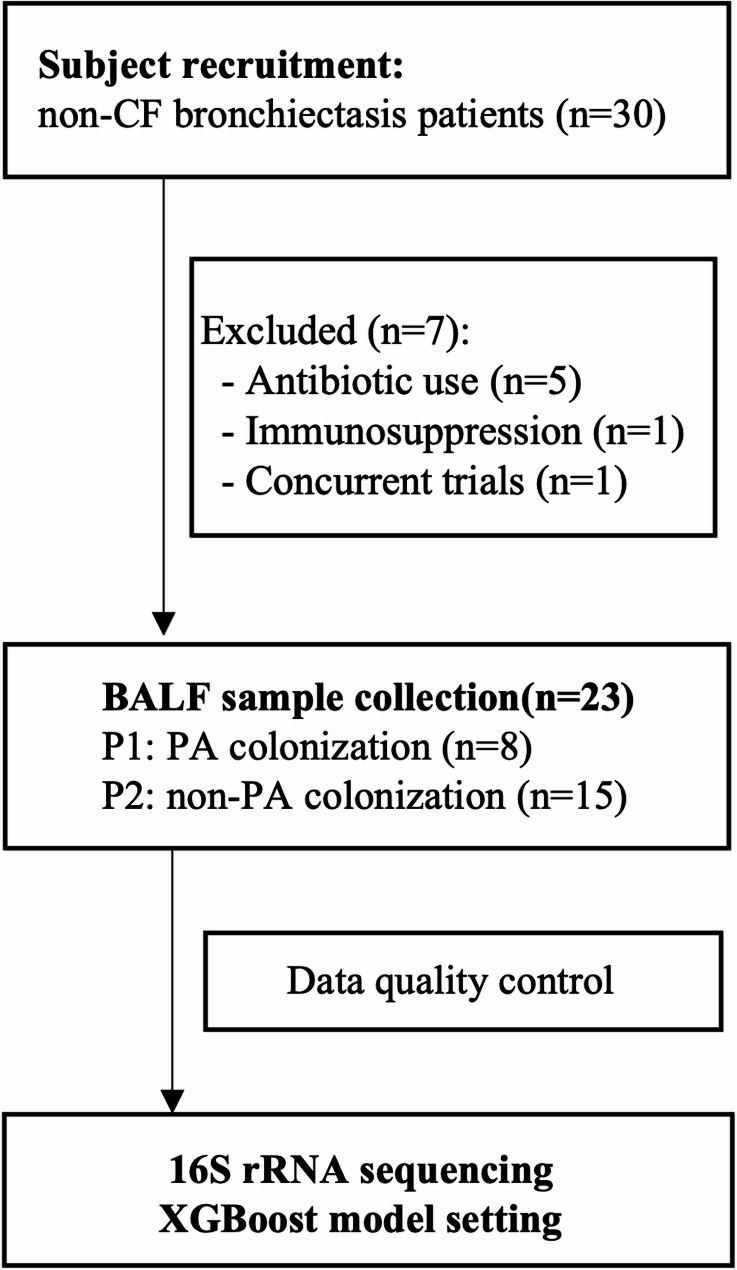
Flowchart. Thirty patients with non-cystic fibrosis bronchiectasis were recruited from Shanghai Pulmonary Hospital with seven excluded based on predefined exclusion criteria. The remaining 23 eligible patients underwent BALF collection for 16 S rRNA gene sequencing

Thirty patients with non-cystic fibrosis bronchiectasis were recruited from the Shanghai Pulmonary Hospital between August 2019 and June 2020, with seven excluded based on predefined exclusion criteria. The remaining 23 eligible patients underwent BALF (bronchoalveolar lavage fluid) collection for further analysis. Inclusion criteria included a radiologically confirmed diagnosis of bronchiectasis via high-resolution computed tomography (HRCT), age ≥ 18 years, and stable clinical status without acute exacerbation within four weeks prior to sample collection. All participants were completely free of antibiotic use including long-term maintenance therapies and prior eradication regimens such as azithromycin or inhaled tobramycin for a minimum of four weeks prior to sample collection. Exclusion criteria comprised cystic fibrosis, active tuberculosis, significant immunosuppression, or concurrent participation in other interventional studies. Specific procedures are detailed in Fig. 1.

### BALF sampling and clinical data collection

BALF samples were obtained via fiberoptic bronchoscopy following standard clinical guidelines [13]. The bronchoscope was wedged into the segmental bronchus of the most radiologically affected lung lobe. A total of 100 mL sterile saline was instilled in 20 mL aliquots and gently aspirated, with the first aliquot discarded to minimize upper airway contamination. Recovered BALF was placed on ice and processed within 2 h.

Clinical data were systematically collected at baseline to assess patient demographics, bronchiectasis severity, inflammatory status, and disease progression. Age, sex, and prior antibiotic use within four weeks were recorded for all participants. Bronchiectasis Severity Index (BSI) was calculated based on clinical parameters, including exacerbation history, radiological findings, and comorbidities. Dyspnea severity was assessed using the modified Medical Research Council (mMRC) scale. Quality of life was assessed using the St. George’s Respiratory Questionnaire (SGRQ) [14]. Inflammatory biomarkers were measured from peripheral blood samples obtained at the time of BALF collection. C-reactive protein (CRP), Serum amyloid A protein (SAA) and erythrocyte sedimentation rate (ESR) were quantified using standard clinical assays. Complete blood count (CBC) was analyzed, including neutrophil, lymphocyte, monocyte, eosinophil, and basophil counts.

Exacerbations were defined according to the British Thoracic Society definition as an acute deterioration with increasing sputum volume and purulence and/or systemic upset [15]. Exacerbation history was documented both retrospectively (prior 12 months) and prospectively (follow-up 12 months). Patients were categorized as PA-colonized (P1) if *Pseudomonas aeruginosa* was isolated from sputum cultures on ≥ 2 occasions (with ≥ 3-month intervals) within the preceding year [6]. Those with consistently negative cultures during the same period were classified as non-colonized (P2). Patients with only one positive culture were excluded to avoid misclassification of transient colonization.

The study was approved by the Ethics Committee of Shanghai Pulmonary Hospital, School of Medicine, Tongji University (approval number K19-144). All procedures involving human participants were performed in accordance with the ethical standards of the institutional research committee and the Helsinki Declaration. Written informed consent was obtained from all individual participants prior to sample collection and data acquisition, with particular attention to explaining the risks and benefits of bronchoscopy procedures.

### DNA extraction and 16 S rRNA gene amplification

Microbial DNA was extracted from bronchoalveolar lavage fluid (BALF) samples using the QIAamp DNA Mini Kit (Qiagen, Germany) following the manufacturer’s protocol [16]. DNA quality and concentration were assessed using a NanoDrop 2000 spectrophotometer (Thermo Fisher Scientific, USA), and DNA integrity was evaluated by 1% agarose gel electrophoresis, revealing intact genomic DNA without smearing or degradation (see Additional File_Supplementary Figure S1). Extracted DNA was stored at −80°C until further analysis. The V3-V4 hypervariable region of the 16S rRNA gene was amplified using universal primers 341 F (5’-CCTACGGGRSGCAGCAG-3’) and 806R (5’-GGACTACVVGGGTATCTAATC-3’). PCR reactions were carried out in triplicate using KAPA HiFi HotStart ReadyMix (Roche, Switzerland) to ensure high fidelity. Amplicons were purified using the AxyPrep DNA Gel Extraction Kit (Axygen, USA) and quantified with Qubit prior to sequencing library preparation.

### Sequencing, bioinformatics processing, and microbial analysis

Sequencing libraries were generated using the Illumina TruSeq DNA Sample Preparation Kit and indexed with Nextera XT adapters. Paired-end sequencing (2 × 250 bp) was performed on the Illumina MiSeq platform (Illumina, USA). Raw sequencing reads were demultiplexed, quality-filtered using Trimmomatic, and processed using the QIIME pipeline (v1.9.1) to generate operational taxonomic units (OTUs) at 97% similarity via UCLUST clustering. While dedicated sequencing negative controls were not included, all BAL procedures utilized sterile saline with DNA-free certified consumables. Bioinformatics pipelines incorporated stringent filtering steps (Q30 quality threshold, N-base removal, length selection) to mitigate contamination risks. Chimeric sequences were removed using the UCHIME algorithm, and taxonomy was assigned based on the SILVA 138 database (97% similarity).

Microbial community diversity was assessed using alpha diversity metrics (Shannon index, Simpson index) and beta diversity (weighted and unweighted UniFrac distances). Principal coordinate analysis (PCoA) and non-metric multidimensional scaling (NMDS) were applied to visualize community differences. Differentially abundant taxa between PA-colonized (P1) and non-colonized (P2) groups were identified with the linear discriminant analysis effect size (LEfSe) algorithm and MaAsLin2 regression, adjusting for age, BSI scores, and inflammatory markers. Functional pathway prediction was conducted using PICRUSt2, mapping microbial gene content to KEGG and MetaCyc databases to infer metabolic potential and host-microbe interactions. Detailed bioinformatics analysis procedures are shown in Additional File_ Supplementary Figure S2.

### Statistical analysis

All statistical analyses were performed using R (v4.3.1). Continuous variables were assessed for normality using the Shapiro-Wilk test, and non-parametric tests (Mann-Whitney U for two groups; Kruskal-Wallis for three or more groups) were applied due to the small sample size. Descriptive statistics for continuous variables were expressed as medians with interquartile ranges (IQR), and categorical variables as frequencies with percentages. Spearman’s rank correlation coefficients were calculated to evaluate relationships between microbial abundances, inflammatory markers and clinical parameters, with correlation heatmaps used to visualize associations. A p-value < 0.05 was considered statistically significant. To address multicollinearity between *Pseudomonas* abundance and BSI scores, sensitivity analyses excluded BSI-related variables from correlation and regression models. Linear regression models were adjusted for age, sex, and prior exacerbation history to assess independent effects of microbial taxa and inflammatory markers on disease severity and exacerbation risk.

### Model development and validation

An XGBoost model was developed to predict 1-year acute exacerbation risk by integrating microbial taxa relative abundances, alpha diversity indices, inflammatory markers and clinical parameters [17]. To avoid multicollinearity, BSI was excluded from the model. Missing data were imputed using the k-nearest neighbor (KNN) algorithm, and the dataset was randomly split into training (80%) and validation (20%) sets. Hyperparameters, including learning rate, maximum tree depth, and boosting rounds, were optimized via nested cross-validation with grid search. Model performance was evaluated using AUC, precision, recall, and F1-score, with SHapley Additive exPlanations (SHAP) analysis employed to interpret feature contributions. Sensitivity analysis assessed the impact of key features (e.g. *Pseudomonas* abundance, CRP levels) on model performance, and bootstrapping (1,000 iterations) was used to estimate metric stability. Calibration plots compared predicted probabilities with observed exacerbation frequencies.

## Results

### Patient characteristics and clinical parameters

A total of 23 non-cystic fibrosis bronchiectasis patients were stratified into two subgroups: 8 with *Pseudomonas aeruginosa* (PA) colonization (P1) and 15 without PA colonization (P2). Demographic characteristics, including age (P1: 48.75 ± 13.13 vs. P2: 47.07 ± 16.80, *P* = 0.820), BMI (21.48 ± 1.80 vs. 21.49 ± 3.32, *P* = 0.989), and sex distribution (male: 25.0% vs. 47.4%, *P* = 0.388), showed no significant intergroup differences (Table 1). Systemic inflammation biomarkers revealed comparable neutrophil counts (4.27 ± 1.33 vs. 4.36 ± 2.21, *P* = 0.095) and erythrocyte sedimentation rate (ESR) levels (59.12 ± 41.08 vs. 35.68 ± 24.30, *P* = 0.075) between groups, though a trend toward elevated ESR was observed in the P1 cohort. C-reactive protein (CRP), serum amyloid A protein (SAA), lymphocyte, monocyte, eosinophil, and basophil counts similarly exhibited no significant differences (all *P* > 0.05).

**Table 1:** Descriptive statistics of study participants

Characteristic	level	P1	P2	*P* value
n		8	15	
Sex ^‡^	Male	2 (25.0)	8 (47.4)	0.388
	Female	6 (75.0)	7 (52.6)	
Age (years) ^†^		48.75 (13.13)	47.07 (16.80)	0.820
BMI (kg/m²) ^†^		21.48 (1.80)	21.49 (3.32)	0.989
FEV1% predicted ^†^		62.69 (21.29)	69.47 (19.39)	0.427
CRP (mg/L) ^†^		7.74 (3.92)	5.48 (2.63)	0.113
SAA (mg/L) ^†^		72.21 (138.86)	48.74 (83.72)	0.616
ESR (mm/h) ^†^		59.12 (41.08)	35.68 (24.30)	0.075
Neutrophil_Count (10^9^ cells/L) ^†^		4.27 (1.33)	4.36 (2.21)	0.095
Lymphocyte_Count (10^9^ cells/L) ^†^		1.74 (0.73)	2.22 (1.07)	0.260
Monocyte_Count (10^9^ cells/L) ^†^		0.46 (0.11)	0.56 (0.27)	0.294
Eosinophil_Count (10^9^ cells/L) ^†^		0.17 (0.12)	0.16 (0.11)	0.747
Basophil_Count (10^9^ cells/L) ^†^		0.02 (0.01)	0.03 (0.02)	0.097
Past_Year_Exacerbations ^†^		3.00 (2.14)	1.20 (1.47)	*
Follow_Up_Year_Exacerbations ^†^		3.75 (2.05)	0.80 (1.15)	***
BSI_Score ^†^		8.38 (4.07)	4.33 (2.32)	**
SGRQ_Score ^†^		35.75 (8.84)	22.79 (5.40)	***
CAT_Score ^†^		24.00 (6.16)	16.26 (5.27)	**
mMRC_Score ^†^		1.62 (0.52)	0.95 (0.62)	*

*Abbreviations*: *BMI* Body mass index, *BSI* Bronchiectasis severity index, *FEV1* Forced expiratory volume in 1s, *CRP* C-reactive protein, *SAA* Serum amyloid A protein, *ESR* Erythrocyte sedimentation rate, *BSI* Bronchiectasis Severity Index SGRQ, St. George’s Respiratory Questionnaire, *CAT* COPD Assessment Test, *mMRC* Modified Medical Research Council (mMRC) scale

^†^mean (SD)

^‡^n (%)

*P < 0.05

**P < 0.01

***P < 0.001

Clinically, P1 demonstrated markedly worse disease severity, as evidenced by higher Bronchiectasis Severity Index (BSI) scores (8.38 ± 4.07 vs. 4.33 ± 2.32, *P* < 0.01), poorer quality-of-life metrics including the St. George’s Respiratory Questionnaire (SGRQ: 35.75 ± 8.84 vs. 22.79 ± 5.40, *P* < 0.001) and COPD Assessment Test (CAT: 24.00 ± 6.16 vs. 16.26 ± 5.27, *P* < 0.01), as well as elevated dyspnea severity assessed by the modified Medical Research Council (mMRC) scale (1.62 ± 0.52 vs. 0.95 ± 0.62, *P* < 0.05). Notably, the P1 group experienced significantly more acute exacerbations both retrospectively (3.00 ± 2.14 vs. 1.20 ± 1.47 events/year, *P* < 0.05) and prospectively (3.75 ± 2.05 vs. 0.80 ± 1.15 events/year, *P* < 0.001) compared to non-colonized patients (Table 1).

### *Pseudomonas*-driven ecological disruption and airway dysbiosis

**Fig. 2:**
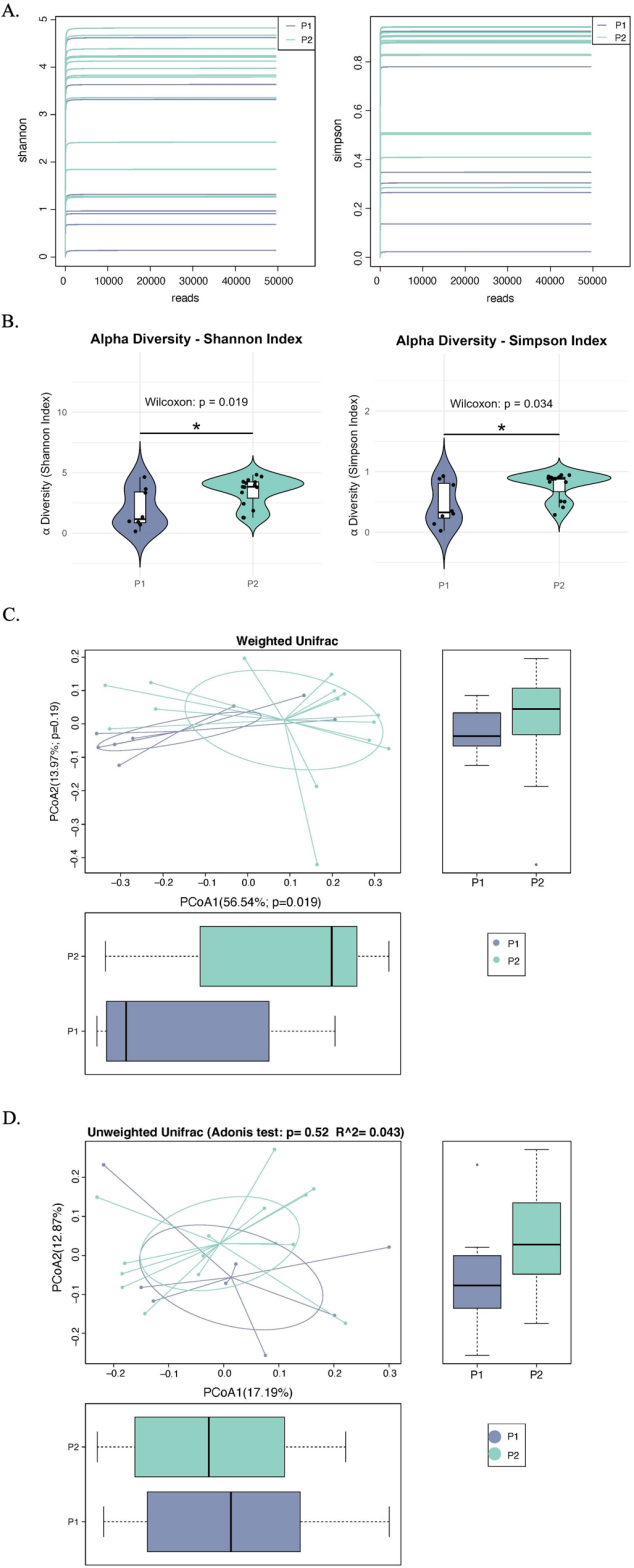
Microbial Diversity and Composition in BALF. **A** Rarefaction curves demonstrating sufficient sequencing depth to capture microbial diversity across PA-colonized (P1, purple) and non-colonized (P2, green) groups. **B** Significant reduction in alpha diversity indices in P1 patients: Shannon index (P1: 1.96 vs. P2: 3.47, *P* < 0.05) and Simpson index (P1: 0.46 vs. P2: 0.77; *P* < 0.05). **C** Weighted UniFrac-based PCoA plot showing distinct clustering of P1 and P2 communities (Adonis R² = 0.162, *P* < 0.05). **D** Unweighted UniFrac PCoA revealing non-significant group separation (*P* > 0.1)

Microbial diversity profiles in bronchoalveolar lavage fluid (BALF) were systematically characterized through alpha and beta diversity analyses. As shown in the rarefaction curves (Fig. 2A), adequate sequencing depth was achieved to comprehensively assess microbial communities across all samples. Comparative analysis revealed significant differences in alpha diversity between patient groups. Specifically, P1 patients demonstrated marked reductions in diversity indices compared to P2 patients, with statistically significant decreases in the Shannon index (*P* < 0.05) and Simpson index (*P* < 0.05) (Fig. 2B). This pattern suggests the emergence of a simplified microbial ecosystem in the P1 group. While other alpha diversity metrics, including the Observed species index, Chao1 index and PD_whole_tree index did not reach statistical significance, they consistently trended lower in P1 (Additional File_Supplementary Figure S3).

Beta diversity analysis further highlighted structural differences between groups. Weighted UniFrac-based PCoA revealed clear separation of microbial communities between P1 and P2 cohorts (Fig. 2C), with Adonis permutation test confirming statistically significant compositional differences (R² = 0.162, *P* < 0.05). Notably, this separation was primarily driven by variations in dominant taxa abundance rather than rare species composition, as evidenced by the non-significant group separation in unweighted UniFrac analysis (Fig. 2D).

The collective findings demonstrate that PA colonization is associated with substantial remodeling of airway microbiota, characterized by two key features: (1) significant depletion of microbial diversity, (2) establishment of a dysbiotic state.

### *Pseudomonas* dominance and commensal ecological shifts in PA-Colonized patients

**Fig. 3:**
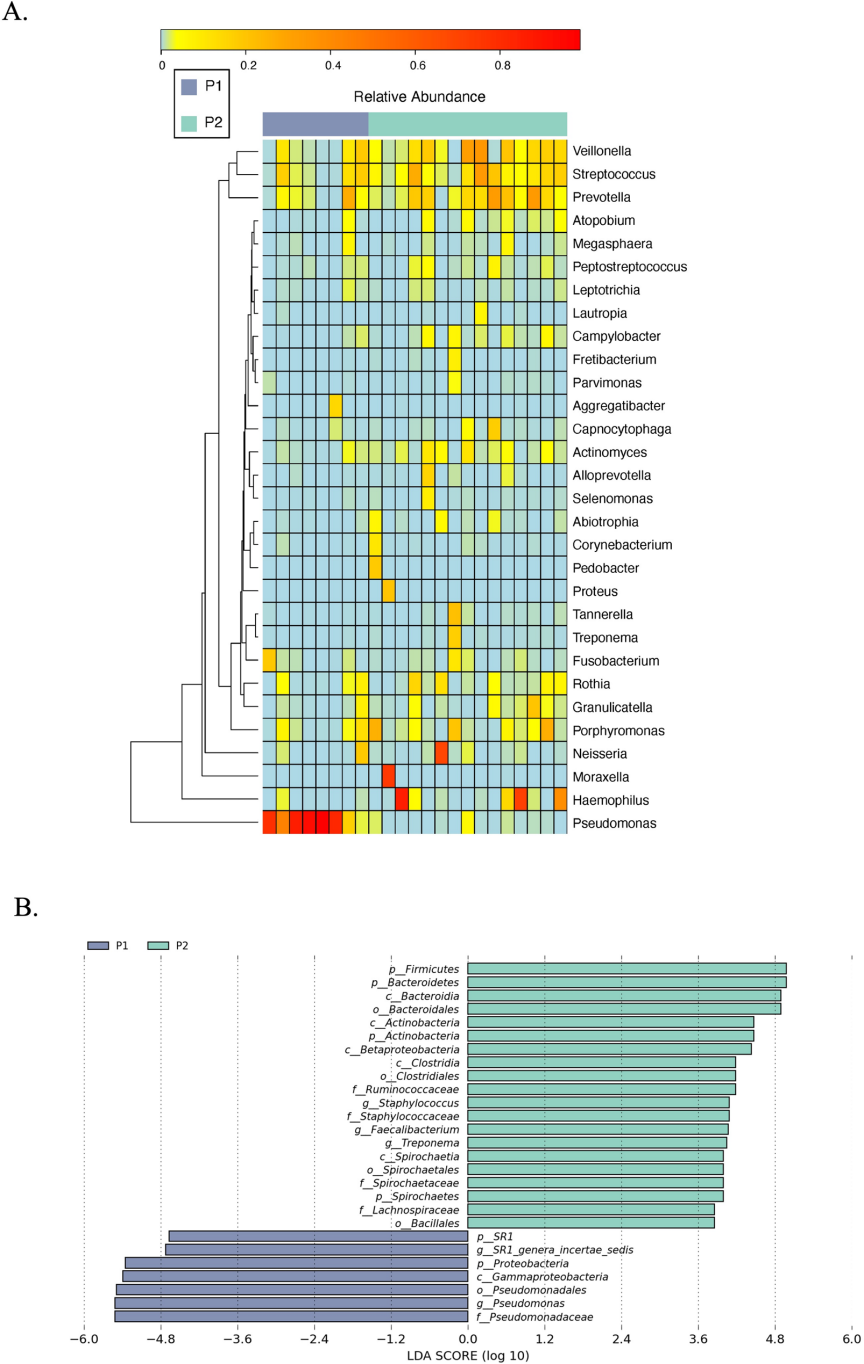
*Pseudomonas* dominance and ecological shifts in bronchiectasis airway microbiota **A** Heatmap of relative microbial abundances in BALF from PA-colonized (P1, *n* = 8) and non-colonized (P2, *n* = 15) patients. Commensal genera (*Veillonella*, *Prevotella*, *Streptococcus*) show higher abundances in P2, while *Pseudomona* dominates the P1 microbiome (all *P* > 0.05 after FDR correction). **B** LEfSe analysis reveals signature microbiome differences in PA-colonized groups. Bar plot shows LDA scores (threshold ≥ 2.0 for significance). *Pseudomonas* (LDA > 4) were significantly enriched in the PA-colonized group (P1), while commensal bacteria (*Veillonella* spp. etc.) showed no significant differences (LDA < 2), suggesting collateral depletion of commensals due to *Pseudomonas* niche competition

Microbial composition analysis revealed distinct taxonomic patterns between P1 and P2 groups. While *Pseudomonas* dominated the P1 microbiota, visualization of relative abundance patterns through heatmap analysis (Fig. 3A) suggested potential ecological differences in commensal genera. Specifically, *Veillonella* (P1:5.1% vs. P2:11.6%), *Prevotella* (P1:5.2% vs. P2:11.8%), and *Streptococcus* (P1:6.4% vs. P2:10.6%) exhibited nominally higher abundances in P2, though these differences did not reach statistical significance (all *P* > 0.05, Mann-Whitney U test with FDR correction).

Notably, LEfSe analysis (Fig. 3B) confirmed these genera were not key discriminators (LDA < 2.0), aligning with their secondary role in community divergence.

### Machine Learning-Based prediction of acute exacerbations

**Fig. 4:**
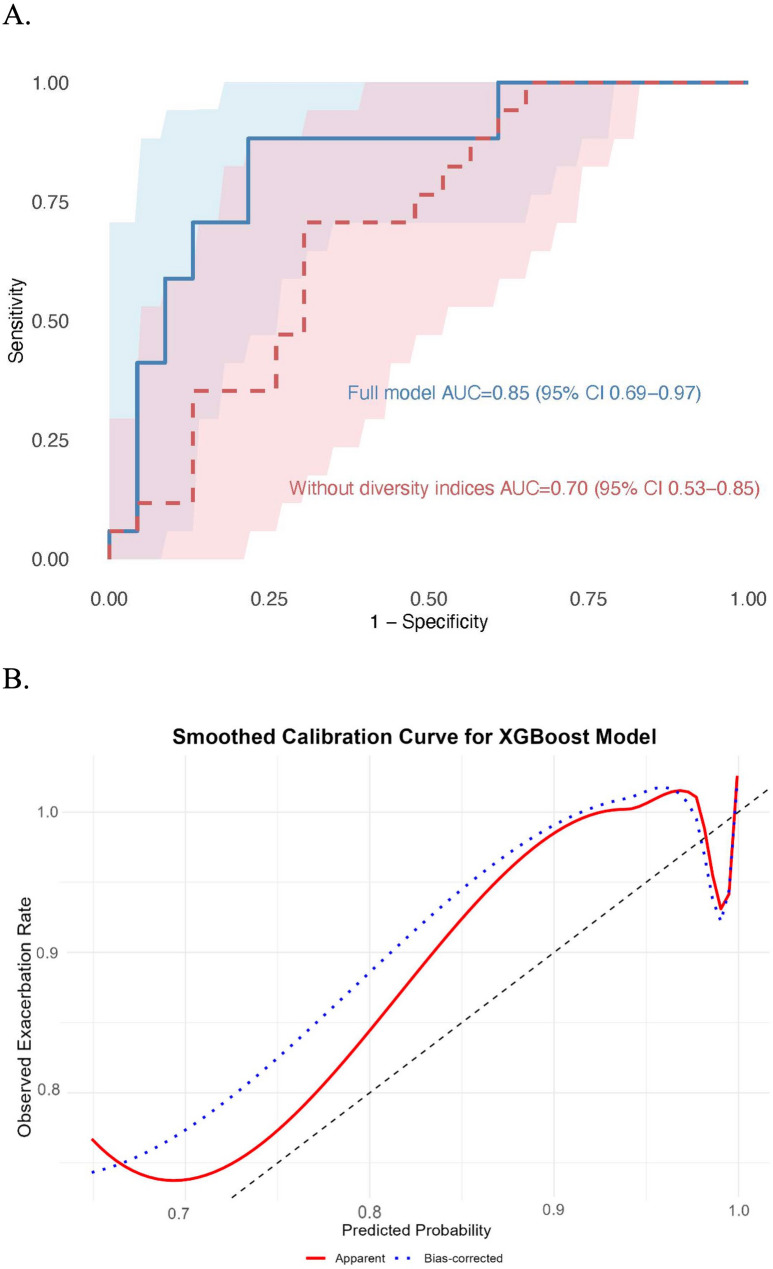
Predictive performance of the XGBoost model for exacerbation risk. **A** ROC curve showing robust discrimination (solid blue lines, AUC = 0.85, 95%CI: 0.69–0.97) and reduced predictive accuracy with the exclusion of microbial diversity indices (dashed red lines, AUC = 0.70, 95%CI: 0.53-085). **B** Calibration curve demonstrating alignment between predicted and observed probabilities

The XGBoost model, integrating microbial, inflammatory, and clinical variables, demonstrated robust performance in predicting 1-year acute exacerbation risk. Key predictors encompassed microbial taxa (*Pseudomonas*, *Prevotella*, *Veillonella*), alpha diversity indices (Shannon and Simpson index), inflammatory markers (CRP, ESR, neutrophil count), and clinical parameters (age, sex, FEV1% predicted, prior exacerbations). The model achieved an AUC of 0.85 (95%CI: 0.69–0.97) (Fig. 4A), with high precision (0.82) and recall (0.87) derived from the confusion matrix at the optimal threshold. Calibration plots showed good agreement between predicted and observed exacerbation probabilities, supporting the model’s reliability (Fig. 4B). The inclusion of microbial diversity and taxa interactions, rather than focusing solely on *Pseudomonas* abundance, highlighted the importance of airway microbiota stability in exacerbation risk prediction.

### Model interpretation and ecological insights

**Fig. 5:**
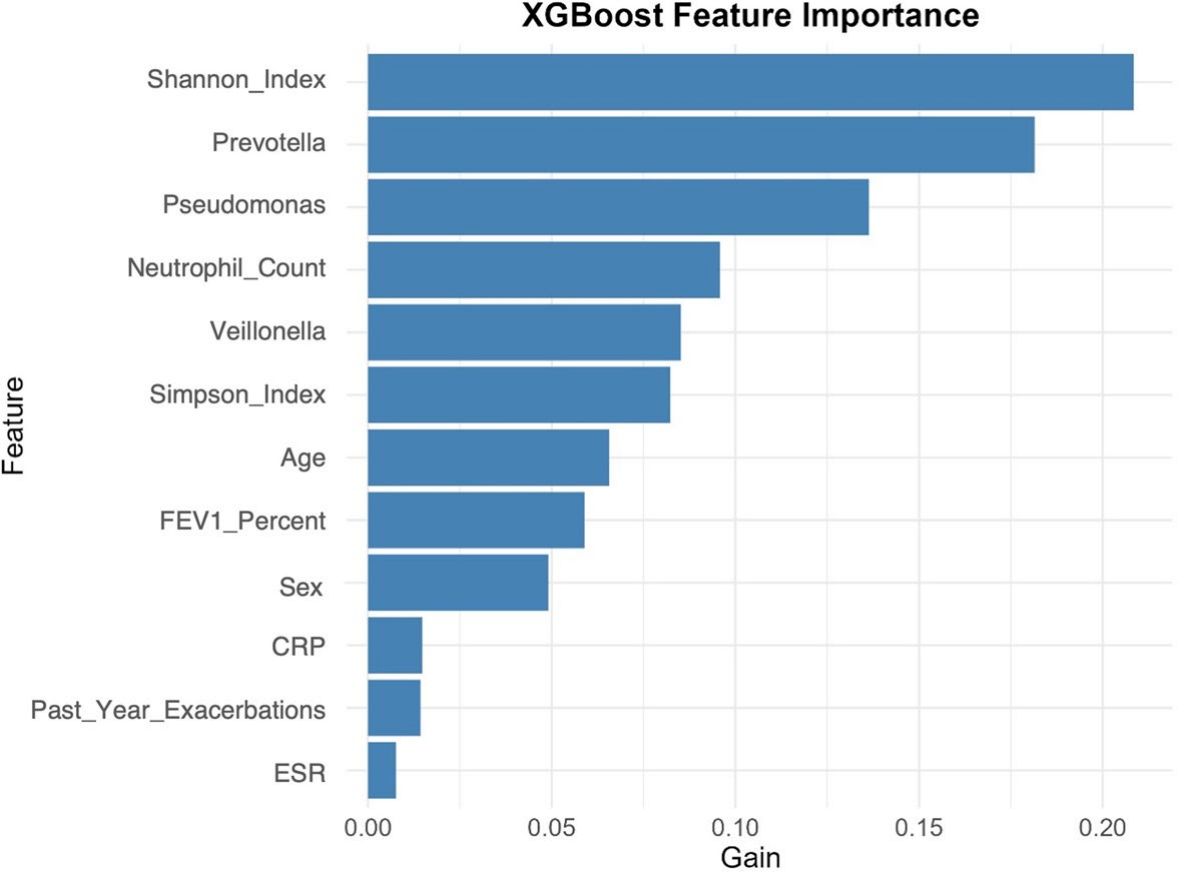
Feature importance in exacerbation risk prediction. Shannon diversity index and *Prevotella* abundance were top predictors, outperforming *Pseudomonas*

SHapley Additive exPlanations (SHAP) analysis identified microbial diversity (Shannon index) and *Prevotella* abundance as the most influential predictors of exacerbation risk (Fig. 5). While *Pseudomonas* contributed to predictions, its impact was subordinate to community-level features, reflecting the ecological destabilization induced by pathogen dominance. Sensitivity analysis further validated model robustness: exclusion of microbial diversity indices reduced predictive accuracy by 15% (Fig. 4, AUC = 0.70 vs. 0.85), highlighting the necessity of incorporating microbiome complexity. These findings collectively emphasize that exacerbation risk arises not from individual pathogens, but from dysbiosis-driven collapse of microbial community resilience.

## Discussion

Bronchiectasis progression is increasingly recognized as a consequence of dynamic host-microbe-environment interactions [2]. Emerging evidence highlights significant dysbiosis in the airways of bronchiectasis patients, characterized by diminished microbial richness and selective depletion of symbiotic taxa—including *Streptococcus*, *Prevotella*, and *Staphylococcus*—alongside disproportionate expansion of opportunistic pathogens such as *Pseudomonas* and *Hemophilus* [18, 19]. This ecological shift is clinically consequential, as reduced microbial diversity in sputum has been consistently linked to accelerated deterioration of pulmonary function [20]. Our study reveals that PA colonization disrupts airway microbial homeostasis not merely through pathogen dominance but by destabilizing the broader ecological network, and reducing microbial diversity. Notably, while Observed Species, Chao1, and PD whole-tree indices showed consistent numerical trends toward reduced diversity in PA-colonized patients (Additional File_Supplementary Figure S3), these differences did not reach statistical significance (all *P* > 0.05). This lack of significance likely reflects limited statistical power due to our modest sample size rather than biological irrelevance, as all three metrics directionally aligned with the significant reductions in Shannon/Simpson indices (Fig. 2B). *Pseudomonas* dominance is related to reduced relative abundances of the commensal genera which are associated with maintaining respiratory microbiome homeostasis: *Veillonella* metabolizes lactate into propionate and acetate, which exhibit antimicrobial properties against pathogens [21]; *Prevotella* modulates mucosal immunity through Th17/Treg balance regulation [22]. In contrast, the dominance of PA in P1 correlated with reduced commensal diversity, likely due to its secretion of antimicrobial compounds (e.g., pyocyanin, rhamnolipids) and niche competition via biofilm establishment [23]. These ecological alterations may create a self-perpetuating cycle that promotes disease advancement and predisposes patients to recurrent exacerbations in bronchiectasis.

Crucially, the influence of antibiotic exposure and comorbid conditions must be contextualized. Prior antibiotic administration may disrupt ecological networks within the lung microbiota [4], reduce microbial diversity [24], and alter P. aeruginosa sputum density [25] – collectively biasing microbiome-derived predictors. Similarly, distinct disease etiologies drive divergent microbial landscapes: Cystic fibrosis (CF) and non-CF bronchiectasis exhibit fundamentally different microbiome architectures and antimicrobial resistance profiles [26]. Tuberculosis infection remodels airway ecology through host immune modulation, enhancing susceptibility to exogenous pathogen colonization [27]. These etiology-specific microbial restructuring processes could confound generalizable bronchiectasis signatures. Consequently, we excluded patients with prior antibiotic use and co-morbidities (e.g. active tuberculosis and cystic fibrosis). Although we excluded patients with recent antibiotic exposure, our study did not capture the potential long-term microbiome alterations induced by historical antibiotic regimens (e.g., repeated eradication courses for PA). Future studies with detailed antibiotic history and longitudinal sampling could clarify these persistent effects.

The potential clinical implications of these observed ecological alterations warrant consideration. Our machine learning framework, integrating microbial diversity indices with inflammatory markers, identifies microbiome resilience—rather than PA abundance—as the strongest predictor of 1-year exacerbation risk. These findings align with emerging evidence highlighting the importance of microbial community resilience in chronic respiratory diseases [28, 29].

The predictive power of our XGBoost model further validates the clinical relevance of microbial community profiling. Notably, SHAP analysis identified microbial diversity and commensal taxa abundances as stronger predictors of exacerbation risk than PA abundance itself, challenging the conventional emphasis on pathogen-centric biomarkers. While microbial diversity emerged as a stronger predictor of exacerbation risk than *Pseudomonas* abundance in our models, it is plausible that the observed reduction in diversity reflects, at least partially, the cumulative ecological impact of chronic PA colonization, consistent with our suggestion that chronic colonization may suppress commensal microbiota. The cross-sectional nature of our microbiome sampling limits our ability to definitively disentangle whether reduced diversity is a cause or a consequence of long-term colonization dynamics. Nevertheless, the superior predictive performance of diversity metrics in our prospective analysis suggests that they capture a clinically relevant state of airway ecosystem dysbiosis. We therefore propose that microbial diversity serves as an integrative biomarker, reflecting both the immediate pathogen burden and the historical trajectory of airway colonization, thus encapsulating aspects of chronicity within its measure. This framework reconciles chronicity concerns with the demonstrated clinical utility of diversity metrics.

Meanwhile, we acknowledge several limitations. First, the most significant limitation of this study remains the relatively small sample size (*n* = 23), which constrains the statistical power and generalizability of our findings. Second, the use of OTU clustering (97% similarity) rather than ASVs limits species-level taxonomic resolution. Third, the absence of sequencing negative controls restricts definitive exclusion of contamination artifacts, though sterile protocols and bioinformatics filters were rigorously applied to mitigate this risk. Finally, long-term antibiotic effects were not captured. Validation in larger cohorts with optimized methodological controls is needed.

While our findings are constrained by the study’s small sample size and single-center design, they provide a foundation for rethinking bronchiectasis management. Future therapeutic strategies might aim to restore microbial diversity rather than solely suppressing PA. Such approaches could break the cycle of dysbiosis and inflammation, offering a path toward precision management in this heterogeneous disease.

## Conclusions

Our cross-sectional study links airway microbiota dysbiosis with current disease severity and exacerbation frequency in bronchiectasis, though its causal role in disease progression requires longitudinal validation. By leveraging machine learning to decode microbial community interactions, we advance a framework for personalized risk prediction that prioritizes ecological resilience over isolated clinical or microbial markers. These insights not only refine our understanding of bronchiectasis pathophysiology but also align with evolving paradigms in microbiome medicine, where ecosystem health defines therapeutic success.

## Supplementary Information

Supplementary Material 1.

## Data Availability

The datasets associated with this study are present in the paper or the Additional File. The 16 S rRNA sequencing datasets generated during this study have been deposited in the NCBI Sequence Read Archive (SRA) under BioProject accession （https://www.ncbi.nlm.nih.gov/bioproject/PRJNA1263394）.

## Abbreviations

AUC: Area under the curve
BALF: Bronchoalveolar lavage fluid
BMI: Body mass index
BSI: Bronchiectasis severity index
CAT: COPD assessment test
CBC: Complete blood count
CRP: C-reactive protein
ESR: Erythrocyte sedimentation rate
FEV1: Forced expiratory volume in 1s
FDR: False discovery rate
HRCT: High-resolution computed tomography
KNN: K-nearest neighbor
LEfSe: Linear discriminant analysis effect size
mMRC: Modified medical research council scale
NMDS: Non-metric multidimensional scaling
PA: Pseudomonas aeruginosa
PCoA: Principal coordinate analysis
PCR: Polymerase chain reaction
SAA: Serum amyloid A protein
SHAP: SHapley additive explanations
SGRQ: St. George’s respiratory questionnaire
UniFrac: Unique fraction metric
XGBoost: eXtreme gradient boosting
