# Supplementary material for: Pseudomonas aeruginosa-driven airway dysbiosis and machine learning prediction of acute exacerbations in non-cystic fibrosis bronchiectasis: a microbial-inflammatory signature approach
Source: BMC Pulm Med. 2025 Sep 1;25:419. doi: 10.1186/s12890-025-03892-7 (PMC12400642; doi:10.1186/s12890-025-03892-7)
Supplement: Supplementary file 1 — Supplementary Material 1. [file 12890_2025_3892_MOESM1_ESM.docx]

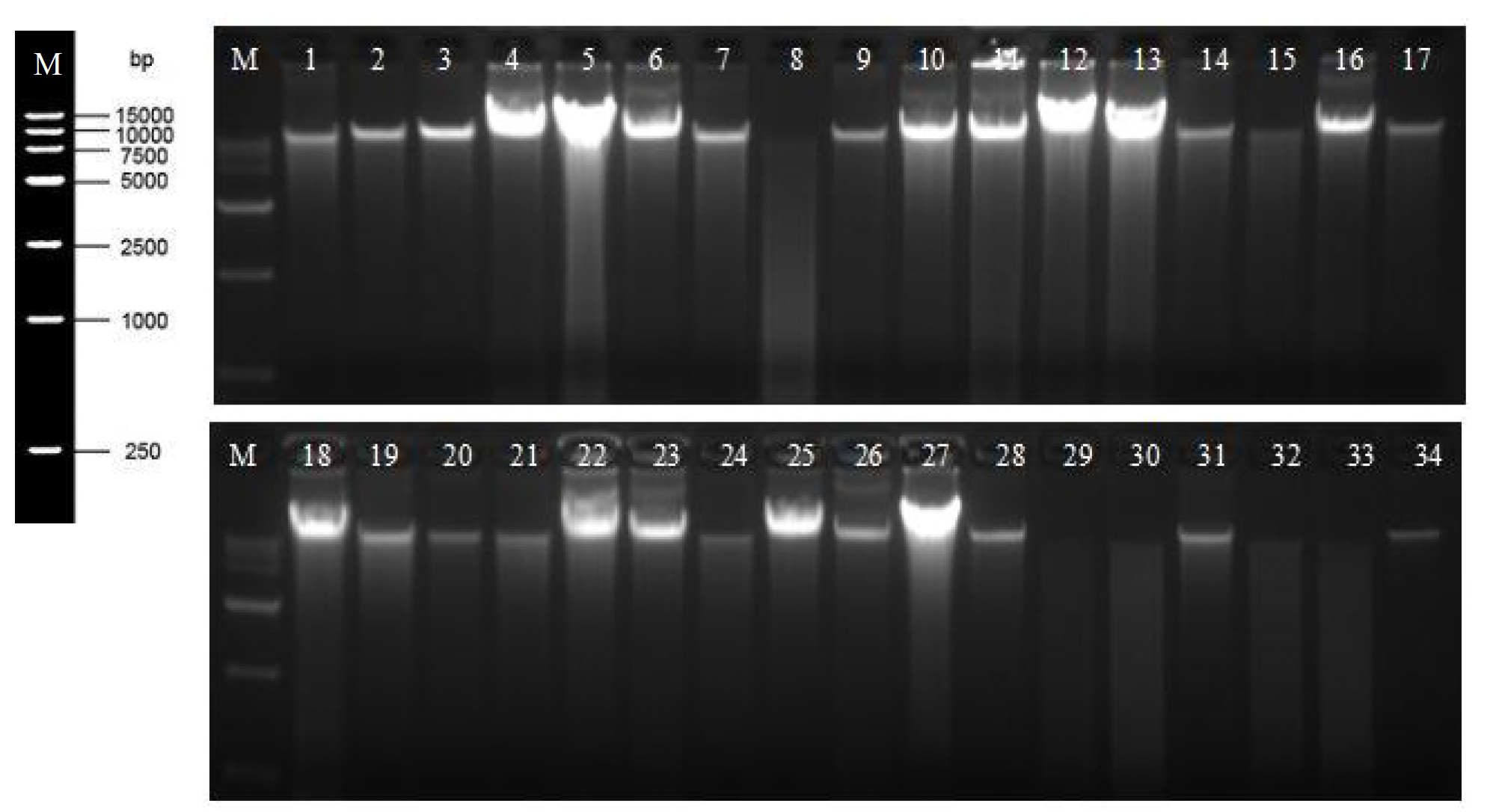


Supplementary Figure S1. DNA gel electrophoresis results.

DNA gel electrophoresis results are presented in the figure, with lanes 1-23 corresponding to BALF-extracted DNA samples from the 23 enrolled non-cystic fibrosis bronchiectasis patients in this study. Agarose gel electrophoresis (1%) was performed for DNA integrity assessment as part of our quality control pipeline prior to 16S rRNA sequencing.

Supplementary Figure S2. Bioinformatics analysis workflow for microbial community characterization.

The pipeline includes raw read processing, quality control, OTU clustering, diversity analyses (alpha: Shannon, Simpson; beta: PCoA, NMDS), species classification, differential species correlation, and functional prediction. Key steps: rarefaction, core microbiome analysis, statistical tests (ANOSIM, MRPP), and environmental factor correlation heatmaps.


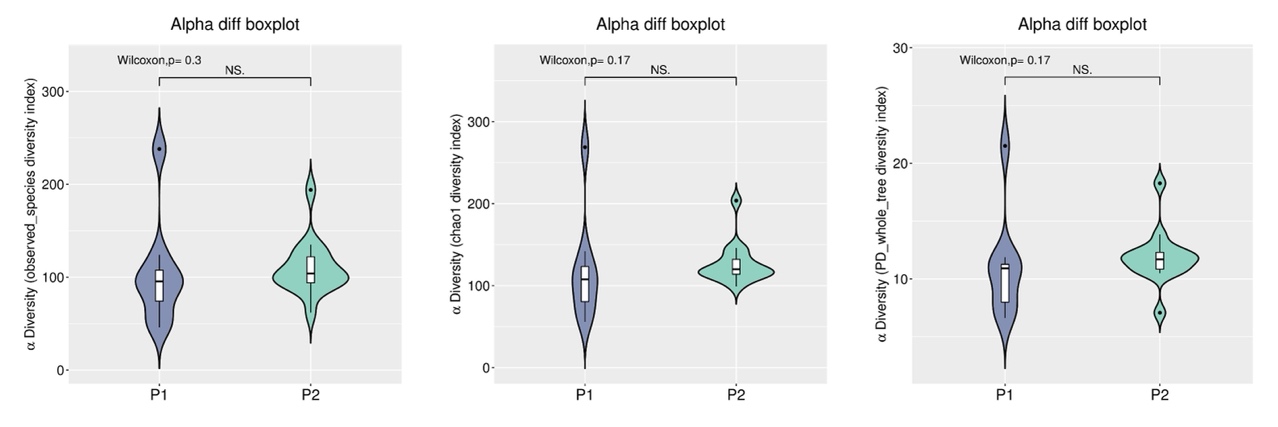


Supplementary Figure S3. Microbial Diversity in BALF.

Alpha diversity indices in bronchiectasis patients: Observed species index (P1: 104.75 vs. P2: 109.93, P = 0.30), Chao1 index (P1: 119.13 vs. P2: 126.67, P = 0.17) and PD_whole_tree index (P1: 11.07 vs. P2: 11.86, P = 0.17).
